# Assessment of Insulin Resistance and Body Composition in Children with Overweight and Obesity: A Pilot Study Using Bioimpedance and Principal Component Analysis

**DOI:** 10.3390/medicina61091709

**Published:** 2025-09-19

**Authors:** Bogdan Mihai Pascu, Anca Bălănescu, Paul Cristian Bălănescu, Corina Delia, Mara Câmpean, Ioan Gherghina

**Affiliations:** 1 Pediatric Department, Faculty of Medicine, University of Medicine and Pharmacy “Carol Davila”, 030167 Bucharest, Romania; anca.balanescu@umfcd.ro (A.B.); paul.balanescu@umfcd.ro (P.C.B.);; 2 National Institute for Mother and Child Health “Alessandrescu-Rusescu”, 020395 Bucharest, Romania; 3 Yuno Clinic, EASO COM Obesity Center, 020459 Bucharest, Romania; 4 Faculty of Chemistry, University of Bucharest, 050663 Bucharest, Romania

**Keywords:** childhood obesity, insulin resistance, bioelectrical impedance analysis, principal component analysis

## Abstract

*Background/Objectives*: Childhood obesity is associated with early metabolic complications, particularly insulin resistance (IR), which significantly elevates the long-term risk for type 2 diabetes and cardiovascular disease. Standard measures such as BMI may inadequately capture metabolic risk, particularly in children with atypical phenotypes such as TOFI (Thin Outside, Fat Inside). This study aimed to evaluate the prevalence and predictors of IR in a pediatric population with overweight and obesity, using both conventional biomarkers and bioelectrical impedance analysis (BIA). We also examined the predictive value of lipid ratios and fasting glucose and applied Principal Component Analysis (PCA) to identify underlying body composition dimensions. *Methods*: A retrospective cohort of 210 children aged 1–18 years, assessed in a tertiary pediatric endocrinology center in Romania, was analyzed. Clinical data included anthropometric measures, fasting laboratory results, and body composition parameters obtained via Tanita PRO DC430 MA BIA. Insulin resistance was defined as HOMA-IR > 2. ROC analysis assessed the predictive performance of triglyceride-to-HDL (Tg/HDL) ratio, fasting glucose, and BIA metrics. PCA was applied to BIA variables to explore dimensional structure. *Results*: Insulin resistance was present in 54.8% of the cohort. It was significantly associated with higher age, pubertal status, ALT, LDL-cholesterol, triglycerides, and BIA-derived fat-free mass (FFM), TBW, and PMM. ROC analyses showed moderate predictive power for Tg/HDL (AUROC = 0.645) and triglycerides (AUROC = 0.656) in identifying IR. BIA metrics had comparable discriminatory performance (AUROC~0.61). PCA reduced eight BIA parameters into two components: a fat-free mass axis (TBW, FFM, PMM, WATERM) and an adiposity axis (BMI, FATP, FATM, WATERP). *Conclusions*: This study highlights the high burden of insulin resistance among children with excess weight and supports the integration of BIA and composite biomarkers into early screening protocols. PCA-derived components may improve metabolic phenotyping in pediatric obesity.

## 1. Introduction

Childhood obesity is a growing public health concern worldwide, associated with an increased risk of metabolic disorders, including insulin resistance (IR), dyslipidemia, and early onset of type 2 diabetes mellitus [1]. Early identification of IR in at-risk pediatric populations remains challenging, especially in those with atypical phenotypes such as TOFI (Thin Outside, Fat Inside). While anthropometric parameters like BMI are commonly used, they may not fully capture metabolic risk. Bioelectrical impedance analysis (BIA) offers a non-invasive method to assess body composition and may provide additional value in evaluating IR risk [2].

This study aimed to assess the proportion and predictors of insulin resistance in children with overweight and obesity using metabolic, anthropometric, and BIA-derived parameters. A secondary objective was to investigate whether new threshold values for the triglyceride-to-HDL cholesterol (Tg/HDL) ratio and fasting glucose levels could act as early red flags for IR in pediatric obesity, prior to the onset of end-organ comorbidities. Additionally, we applied ROC curve and Principal Component Analysis to explore the discriminative and dimensional structure of BIA parameters in relation to metabolic risk and obesity phenotypes. As a pilot investigation, this study also served to map the metabolic and body composition characteristics of the pediatric population affected by obesity attending our medical practice, providing an initial framework for future longitudinal assessments.

## 2. Materials and Methods

### 2.1. Study Population

To address these objectives, we conducted a retrospective pilot study at the National Institute for Mother and Child Health (INSMC) “Alessandrescu-Rusescu”, a public pediatric hospital in Bucharest, Romania. A complete enumeration of all consecutive pediatric patients evaluated in the Department of Pediatric Endocrinology, INSMC “Alessandrescu-Rusescu”, was performed. Medical records spanning a one-year period (1 July 2023–1 July 2024) were screened using the diagnosis-keywords “obesity” or “overweight”, as well as the ICD-10 code E66.0, in order to identify eligible cases for this pilot analysis. Data were extracted from the hospital’s digital medical archive via its electronic health records system. At the time of data extraction, we included all patients aged 1 to 18 years, hereafter referred to as children and adolescents, in line with WHO age group definitions [3]. After de-duplication of multiple visits, a single index encounter per patient was retained. An anonymized dataset containing all variables used for analysis is provided in the Supplementary Materials (Dataset S1). All 210 patients identified through the informatic search had been clinically assessed in the Department of Pediatric Endocrinology, either during hospitalization or in outpatient care. None of the patients were receiving lipid-lowering or glucose-lowering medications. Cases of iatrogenic, genetic, or overt endocrine-related obesity were excluded. Because this was a retrospective pilot study designed to assess feasibility and generate estimates to inform a future powered study, we did not perform a formal a priori power calculation. This represents a pragmatic, complete-enumeration pilot study, intended to evaluate feasibility and generate preliminary estimates to inform the design of a future powered study, rather than to formally test hypotheses.

### 2.2. Laboratory

All patients underwent a standardized laboratory assessment protocol in accordance with hospital guidelines. Blood samples were collected at admission following a minimum fasting period of 8 h. None of the participants were under chronic pharmacological treatment, such as oral antidiabetic or lipid-lowering agents. The baseline laboratory evaluation included complete blood count, fasting blood glucose, liver function tests, urea, creatinine, and lipid profile, and was identically performed for all subjects. Blood analyses were immediately conducted using the Siemens Dimension RXL Max analyzer with commercial reagent kits.

Additional laboratory tests were performed in selected patients based on age, comorbidities, or the primary reason for medical evaluation. These included thyroid function tests (TSH), fasting insulin, glycated hemoglobin (HbA1c), and assessment of the gonadal axis (LH, FSH, SHBG, testosterone, and estradiol). For hormone assays, serum samples were stored at −80 °C and analyzed within a maximum of 7 days using the Cobas U411 platform with standardized commercial kits.

An additional lipid-derived index—the triglyceride-to-HDL cholesterol ratio (Tg/HDL)—was calculated, as it is a well-established marker associated with insulin resistance and atherosclerosis, and thus with increased cardiometabolic risk, in both adults and children. A cut-off value of > 2.73 was used to indicate the presence of insulin resistance, as proposed by Oliveira et al. in 2013 [4].

HOMA-IR was calculated using the formula: fasting insulin (μU/mL) × fasting glucose (mg/dL)/405. A cut-off value of > 2 was used to define insulin resistance. This threshold was chosen based on clinical observations linking it to the presence of metabolic comorbidities, and due to the heterogeneity in age within the cohort. Further stratification by sex or pubertal status would have reduced statistical power, given the limited sample size; therefore, a unified cut-off of 2 was deemed appropriate for this analysis. Moreover, the 2.0 to 2.4 range values are used to define metabolic syndrome criteria [5] and an early detection of IR is always desirable.

### 2.3. Anthropometric Measurements and Body Composition Analysis

In accordance with hospital guidelines, all patients had their anthropometric measurements—height and weight—recorded upon admission. Weight was measured using an electronic medical scale (Seca 799, Seca GmbH & Co. KG, Hamburg, Germany), with children standing still and wearing light clothing. For children under 18 months of age, weight was assessed using an electronic infant scale (Laica PS3001, Laica S.p.A., Vicenza, Italy), with the child undressed and seated. Height was measured using a wall-mounted stadiometer for children able to stand, while recumbent length was recorded using a pediatric measuring board for those under 2 years of age. Body mass index (BMI) was calculated and expressed both as age- and sex-specific percentiles and as standard deviation scores.

Overweight was defined as a BMI percentile between the 85th and 94th centiles for age and sex, while obesity was defined as a BMI percentile above the 95th. In our retrospective cohort, we also identified five subjects with a normal BMI percentile (between the 5th and 85th) who were not excluded, as they demonstrated significant visceral fat accumulation on bioimpedance analysis (visceral fat percentage > 20%) or had a waist-to-height ratio exceeding 0.5.

All patients were evaluated within the Pediatric Endocrinology Department; however, due to the mixed nature of the cohort (inpatients and outpatients), only 127 participants underwent body composition assessment using the Tanita PRO DC430 MA device, Tanita Corporation, Tokyo, Japan. Additional reasons for exclusion from bioelectrical impedance analysis included the age limit (minimum 5 years) and limited cooperation related to medical or neurodevelopmental conditions (e.g., respiratory failure, autism spectrum disorders).

For body composition analysis, children were lightly clothed, asked to void prior to measurement, and stood barefoot on the Tanita PRO DC430 MA device (Tanita Corporation, Tokyo, Japan), ensuring full contact with the metal electrodes. Measurements were conducted at 50 Hz after inputting the subject’s sex, age, and height. The following parameters were recorded: fat percentage (FATP), fat mass (FATM), fat-free mass (FFM), total body water (TBW), predicted muscle mass (PMM), body mass index (BMI), water mass (WATERM), and water percentage (WATERP), as provided by the Tanita DC430 MA system.

### 2.4. Statistical Analysis

Data analysis was conducted using IBM SPSS Statistics for Mac, version 28.0.0, IBM Corp., Armonk, NY, USA. Continuous variables with a normal distribution were presented as means with standard deviations, while non-normally distributed variables were expressed as medians with corresponding minimum and maximum values. Categorical variables were reported as percentages. Differences between groups for non-normally distributed continuous variables were assessed using the Mann–Whitney U test. Receiver Operating Characteristic (ROC) curve analyses was employed to evaluate the discriminative capacity of TBW for identifying insulin resistance as defined by HOMA-IR > 2. Differences in categorical variable distributions were analyzed using chi-square tests. A *p*-value of less than 0.05 was considered statistically significant.

### 2.5. Principal Component Analysis (PCA)

Principal Component Analysis (PCA) was used to reduce dimensions of the bioelectrical impedance analysis (BIA) parameters—FATP, FATM, FFM, TBW, PMM, WATERM, WATERP, and BMI, as all these parameters were intercorrelated. Kaiser-Meyer-Olkin (KMO) measure and Bartlett’s test of sphericity was used to confirm the adequacy of the sample. As data were extractable, Principal Component Analysis (PCA) was subsequently performed using Varimax rotation to extract interpretable components. Components were extracted using a threshold for eigenvalue of 1.

### 2.6. Ethical Consideration

The study was conducted in accordance with the principles of the Declaration of Helsinki and approved by the Ethics Committee of the National Institute for Mother and Child Health “Alessandrescu-Rusescu” (approval code no. 52/03.01.2023). Given the retrospective nature of the study and the use of anonymized data extracted from electronic medical records, no new informed consent was required.

During the preparation of this manuscript, the authors used ChatGPT (OpenAI, San Francisco, CA, USA, GPT-5, 2025) to assist with summarization of results, refinement of English phrasing, and supporting drafting. Access mode: via OpenAI ChatGPT Plus (web interface). No AI use: for data analysis, statistical computation, or figure generation. All AI-assisted content was reviewed and edited by the authors, who take full responsibility for the content of this publication.

## 3. Results

A total of 210 medical records were included in the final analysis. Due to the retrospective nature of the data collection, some records contained missing information. The characteristics of the study cohort are summarized in Table 1.

As with most retrospective studies, a key limitation of this analysis was the incompleteness and heterogeneity of available data. Not all patients underwent the full panel of laboratory investigations required for comprehensive statistical analysis. Therefore, the following section summarizes the subset of data that was available, although not for all 210 overweight or with obesity patients.

Out of the total cohort, only 36 patients had HbA1c measured. Among them, 10 had results within the normal range, 22 (61.1%) had impaired glucose tolerance (HbA1c between 5.5% and 6.5%), and 4 (11.1%) had overt type 2 diabetes mellitus (HbA1c > 6.5%).

Gonadotropin levels were assessed in 107 patients. Based on LH levels exceeding 0.3 mIU/mL—used as a marker for pubertal onset—82 of these patients (76%) had entered puberty.

Thyroid function was assessed in 193 subjects. Among them, 40 had hyperthyrotropinemia (TSH above the age-specific reference range), corresponding to a proportion of 20.7%. Only 2 (1%) of them had suppressed TSH level and the rest of them (151, 78.2%) had normal TSH level.

Regarding the proportion of overweight versus obesity in the subgroup assessed by Tanita device, based on BMI adjusted for age and gender, 100 subjects (78.4%) were classified as living with obesity, 22 (17.32%) as overweight, and 5 (3.9%) fell within the normal weight category. We chose not to exclude these five subjects, as their elevated adipose mass suggested the presence of the ‘TOFI’ phenotype (Thin Outside, Fat Inside), indicating a form of metabolically normal-weight with obesity children.

### 3.1. Insulin Resistance Subgroup Analysis

The proportion of insulin resistance, defined as HOMA-IR > 2, was 54.8% (115 out of 210 subjects).

Compared to the remainder of the cohort (n = 95) without insulin resistance, the insulin-resistant group showed statistically significant associations with higher age (*p* < 0.001), elevated blood glucose levels (*p* = 0.001), increased ALT (*p* = 0.027), higher LDL-cholesterol (*p* = 0.014), and lower HDL-cholesterol (*p* = 0.003). In addition, higher LH levels (*p* = 0.025), elevated triglycerides (*p* < 0.001), increased Tg/HDL ratios (*p* = 0.001), as well as greater weight (*p* = 0.040), BMI (*p* = 0.038), FFM (*p* = 0.039), TBW (*p* = 0.027), PMM (*p* = 0.024), and WATERM (*p* = 0.031) levels were noted. A trend toward statistical significance was also observed for lower testosterone levels (*p* = 0.052) in the insulin-resistant subgroup.

Interestingly, although absolute BMI values were associated with HOMA-IR > 2, there was no significant difference in BMI percentile distribution between insulin-resistant and non–insulin-resistant subjects. Likewise, no statistically significant association was found between HOMA-IR and WATERP levels within the insulin-resistant subgroup (Mann–Whitney U tests).

Chi-square tests were used to evaluate differences between the insulin-resistant and non–insulin-resistant subgroups. No significant differences were found in gender distribution (*p* = 1.0) or urban vs. rural residence (*p* = 0.66). Fasting blood glucose levels did not differ significantly between the two groups (*p* = 0.58).

However, a statistically significant difference was observed with respect to pubertal status: 51 (62.8%) of pubertal subjects had insulin resistance, compared to only 8 (32%) among those without signs of puberty (OR = 3.49, 95% CI: 1.35–9.05).

### 3.2. TOFI Subgroup Descriptive Analysis

A total of five children were classified as having normal weight based on their BMI. However, body composition analysis revealed an increased fat mass percentage, qualifying them as living with obesity according to adiposity criteria. This phenotype, often described as “thin outside, fat inside” (TOFI), is increasingly recognized in pediatric populations. The characteristics of this subgroup are presented below.

Three out of the five were girls, with a median age of 15.5 years (range: 13–17 years). Despite having a normal BMI, three of the children were already insulin resistant. All five had fasting blood glucose levels below 100 mg/dL, with a median of 83 mg/dL (range: 78–98 mg/dL). One girl presented with serum ALT levels approximately twice the upper limit of normal, suggesting a high likelihood of MASLD in the context of insulin resistance.

All children were within the pubertal age range and had normal TSH levels.

The descriptive characteristics of TOFI subgroup are listed in Table 2.

None of the subjects had HbA1c measurements available, and lipid profiles were only tested in two of the five children.

### 3.3. Receiver Operating Characteristic (ROC) Analysis

**Figure 1:**
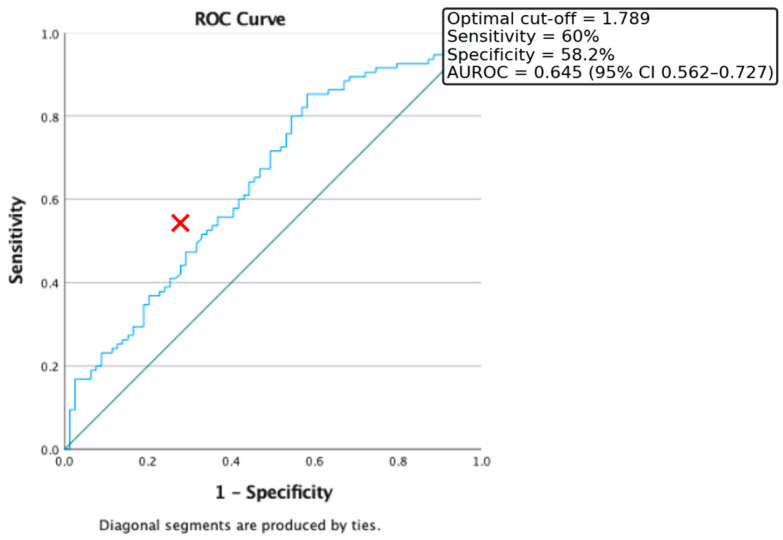
ROC Curve for the discriminative capacity of Tg/HDL Ratio to predict the presence of Insulin Resistance. Red cross represents the optimal cut-off value.

Receiver Operating Characteristic (ROC) curve analysis was conducted to evaluate the discriminative capacity of the triglyceride-to-HDL cholesterol (Tg/HDL) ratio for identifying insulin resistance, defined as HOMA-IR > 2. In our study population, the optimal cut-off value for the Tg/HDL ratio was 1.789, providing a sensitivity of 60% and a specificity of 58.2%. The area under the ROC curve (AUROC) was 0.645 (95% CI: 0.562–0.727) (Figure 1).

**Figure 2:**
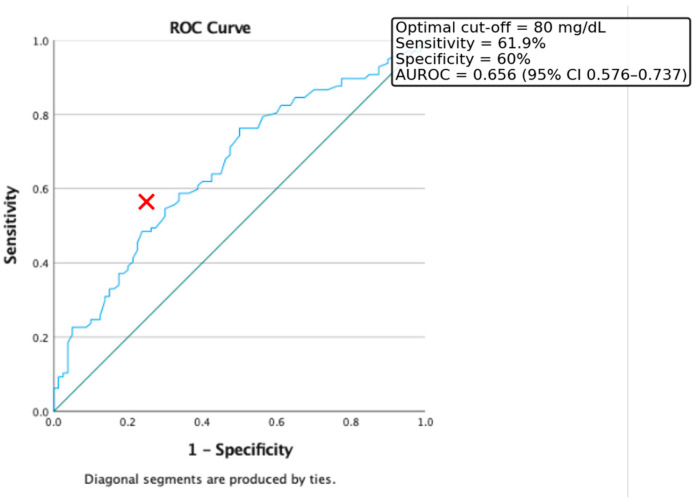
ROC curve for the discriminative capacity of Triglyceride level to predict the presence of Insulin Resistance. Red cross represents the optimal cut-off value.

Additionally, ROC curve analysis was conducted to evaluate the predictive value of triglyceride levels alone in identifying insulin resistance. In our cohort, the optimal cut-off point was 80 mg/dL, yielding a sensitivity of 61.9% and a specificity of 60%. The area under the ROC curve (AUC) was 0.656 (95% CI: 0.576–0.737) (Figure 2).

**Figure 3:**
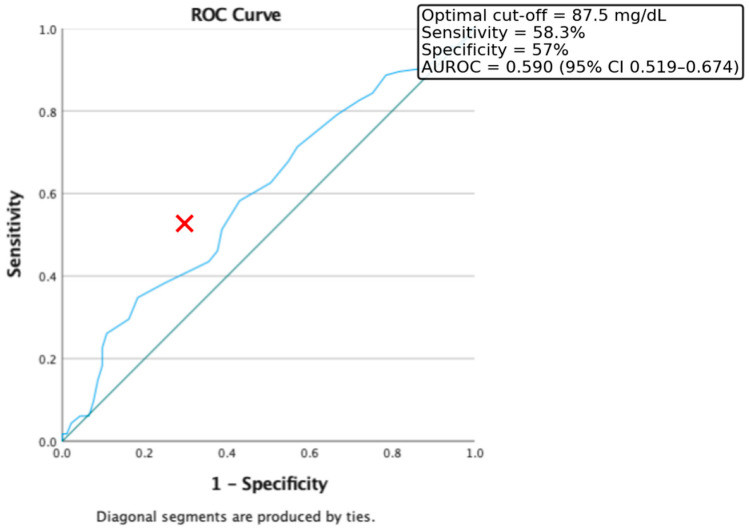
ROC Curve for the discriminative capacity of fasting glucose level to predict the presence of Insulin Resistance. Red cross represents the optimal cut-off value.

Furthermore, ROC curve analysis was used to evaluate the discriminative power of fasting blood glucose levels in identifying insulin resistance, defined as HOMA-IR > 2. In our study population, the optimal threshold was 87.5 mg/dL, which provided a sensitivity of 58.3% and a specificity of 57%. The area under the ROC curve (AUROC) was 0.59 (CI = 0.519–0.674) (Figure 3).

**Figure 4:**
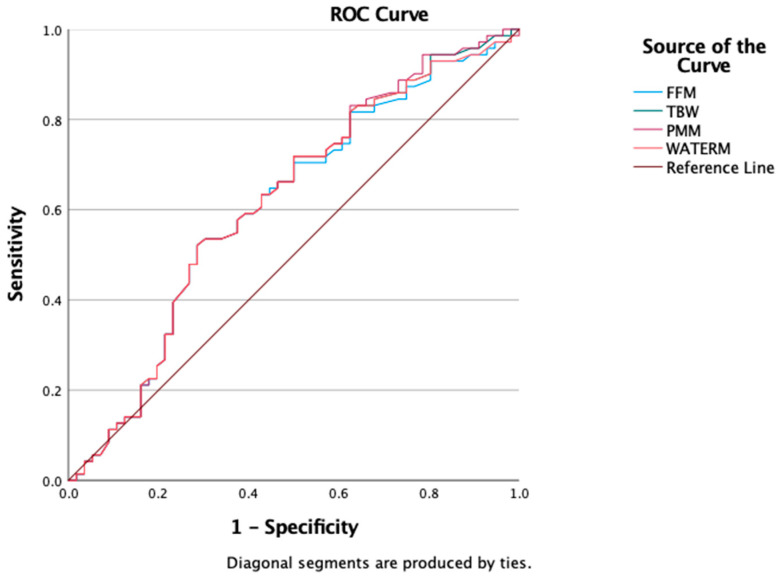
ROC curve for FFM, TBW, PMM, WATERM as a predictors of insulin resistance presence.

We also evaluated the predictive value of BIA-derived parameters (FFM, PMM, TBW, and WATERM) using ROC curve analysis to assess their ability to discriminate insulin resistance. The results showed comparable predictive capacities, with AUROC values around 0.6: FFM = 0.607 (95% CI: 0.506–0.708), TBW = 0.614 (95% CI: 0.514–0.715), PMM = 0.617 (95% CI: 0.517–0.717), and WATERM = 0.612 (95% CI: 0.511–0.712) (Figure 4).

**Figure 5:**
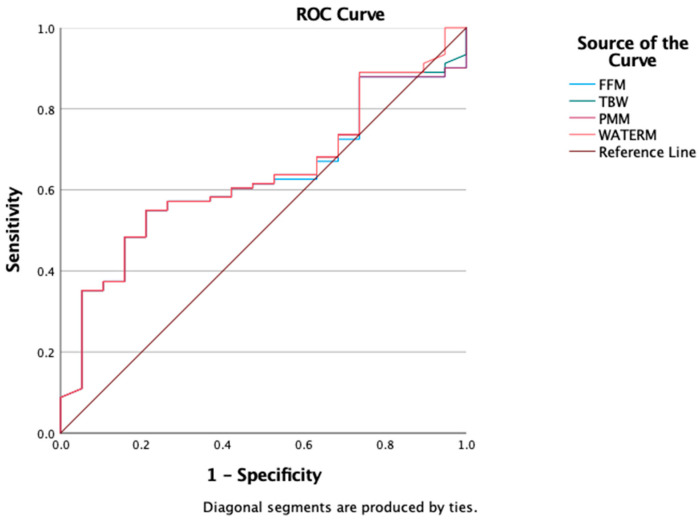
ROC Curves for FFM, TBW, PMM, WATERM as a predictors of obesity.

Finally, ROC curve analysis was conducted to evaluate the discriminatory capacity of bioimpedance analysis (BIA) parameters—total body water (TBW), fat-free mass (FFM), predicted muscle mass (PMM), and water mass (WATERM)—in distinguishing obesity from overweight as defined by BMI. In our cohort, all four parameters showed comparable AUROC values: FFM = 0.607 (95% CI: 0.506–0.708), TBW = 0.614 (95% CI: 0.514–0.715), PMM = 0.617 (95% CI: 0.517–0.717), and WATERM = 0.612 (95% CI: 0.511–0.712) (Figure 5).

Additionaly, three independent variables were included in a binary logistic regression model: fasting glucose (GLU), age, and the triglyceride-to-HDL cholesterol ratio (Tg/HDL). The model revealed that all three predictors were significantly associated with the outcome variable (insulin resistance). Specifically, higher fasting glucose levels were associated with increased odds of the outcome (B = 0.041, *p* = 0.032; OR = 1.042, 95% CI: 1.004–1.081). Age also emerged as a significant predictor (B = 0.184, *p* < 0.001; OR = 1.202, 95% CI: 1.082–1.334), indicating that each additional year of age was associated with a 20% increase in the odds of the condition. Furthermore, the Tg/HDL ratio showed a significant positive association (B = 0.300, *p* = 0.027; OR = 1.349, 95% CI: 1.035–1.760), suggesting that a higher atherogenic index may serve as an independent predictor in this model. The overall model was statistically significant and supports the role of these metabolic parameters in identifying individuals at higher risk.

To consolidate the information from multiple ROC analyses and facilitate direct comparison of predictors, we summarized the AUROC values, optimal cut-offs, and corresponding sensitivity and specificity estimates in Table 3.

### 3.4. Principal Component Analysis

To further explore the collected data, we evaluated the potential correlations among BIA parameters obtained through the Tanita device. Since all parameters demonstrated intercorrelations (see Table 4–Correlation Matrix), we considered the application of Principal Component Analysis (PCA). The suitability for PCA was confirmed by a Kaiser-Meyer-Olkin (KMO) value of 0.643 and a statistically significant Bartlett’s test of sphericity (*p* < 0.05).

**Figure 6:**
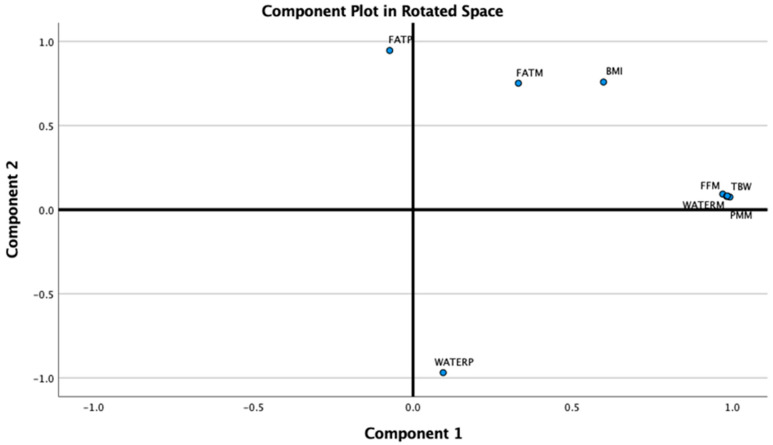
BIA parameters loading the 2 components of the PCA (BIA = Bioimpedance analysis, PCA = Principal Component Analysis).

PCA was then performed using Varimax rotation. The analysis reduced all BIA parameters into two principal components (dimensions) (See Figure 6):

Component 1 (Fat-Free Mass), with an Eigenvalue of 4.77, accounted for 59.61% of the total variance and included TBW (Total Body Water), WATERM (Water Mass), PMM (Predicted Muscle Mass), and FFM (Fat-Free Mass).

Component 2 (Adiposity)**,** with an Eigenvalue of 2.58, explained an additional 32.2% of the variance and included WATERP (Water Percentage), FATP (Fat Percentage), BMI, and FATM (Fat Mass).

## 4. Disscusions

This study addresses the complex challenge of early insulin resistance detection in children with overweight and obesity, including those with atypical phenotypes such as TOFI. By integrating metabolic, anthropometric, and BIA-derived parameters, we aimed to identify early predictors of IR and assess the potential of alternative markers—such as the Tg/HDL ratio and fasting glucose—as early warning indicators before overt metabolic complications arise.

Our findings highlight a high proportion of insulin resistance among Romanian children affected by overweight or obesity, with more than half of the cohort (54.8%) exceeding the HOMA-IR threshold of 2. This finding aligns with global reports indicating that up to 60% of children with obesity may present with IR, especially in tertiary care cohorts and pubertal groups [6,7]. The observed association with pubertal status (OR = 3.49) reinforces existing knowledge that puberty is a period of transient physiological insulin resistance due to hormonal surges in growth hormone and sex steroids [8,9]. Whether puberty-related insulin resistance (IR) alone accounts for some adolescents being classified within the metabolically healthy obesity phenotype, or whether additional concurrent factors contribute to metabolic complications in others (i.e., the metabolically unhealthy obesity phenotype), remains a question requiring further investigation—especially considering the long-term clinical outcomes. While it is well established that all adolescents undergo a degree of physiological insulin resistance during puberty, this should not necessarily raise concern. However, it is equally important that we maintain vigilance in monitoring IR levels, as some individuals may progress to end-organ complications depending on their unique constellation of risk factors. These results underscore the importance of screening for IR around pubertal onset, even in the absence of overt metabolic disease. From a mechanistic perspective, insulin resistance in pediatric obesity is mediated not only by adiposity per se but also by adipose tissue dysfunction, with impaired adipokine secretion, ectopic fat deposition, and systemic low-grade inflammation acting synergistically [10]. An important methodological consideration is our use of a single HOMA-IR threshold (>2) across the entire pediatric cohort. While this cut-off is supported by prior literature as an early marker of metabolic dysfunction, it does not account for age- or puberty-specific variability in insulin sensitivity. Given the heterogeneity of our population (ages 1–18 years), this approach may have led to misclassification in certain subgroups, particularly prepubertal children. We acknowledge that more refined thresholds, stratified by pubertal stage or sex, would be preferable but were not feasible in this pilot study due to sample size limitations. Similarly, the cut-off points derived from our ROC analyses (Tg/HDL ratio, triglycerides, fasting glucose) should be interpreted as exploratory. These values offer preliminary insights for hypothesis generation but are not intended as definitive diagnostic criteria.

Furthermore, we observed that children with IR exhibited significantly higher ALT, triglycerides, LDL-cholesterol, and LH levels, along with lower HDL-cholesterol. These markers are well-established components of the metabolic syndrome and are consistently associated with IR in pediatric populations [11,12]. Elevated ALT may reflect early stages of metabolic dysfunction-associated steatotic liver disease (MASLD), which is increasingly recognized as a hepatic manifestation of pediatric metabolic syndrome [13]. The significant association with ALT in our cohort further supports the hypothesis that hepatic steatosis is an early pathophysiological link between insulin resistance and progression toward MASLD in children. The lack of a significant difference in fasting insulin levels between IR and non-IR groups highlights its limited utility as a standalone marker, and supports the use of integrative indices like HOMA-IR. The inverse correlation between HDL-cholesterol and IR supports its role as a protective factor in cardiometabolic risk stratification [14].

Nevertheless, the Tg/HDL ratio used with a lower threshold than the classic 2.73 proposed by Oliveira et al. in 2013 (cut-off 1.789, AUROC = 0.645) and triglyceride level (cut-off 80 mg/dL, AUROC = 0.656) showed moderate predictive power for identifying IR. These findings are consistent with previous studies suggesting that a Tg/HDL ratio > 2.0 is associated with increased cardiometabolic risk and can serve as a surrogate for IR, particularly in youth [4,15]. Moreover, earlier studies of the authors’ showed that triglyceride levels are independently associated with MASLD (an end-organ disease of insulin-resistance chronic persistence) recommended since 2018 early triglyceride testing (even below the age of ten) in selected patients (overweight and with obesity young children with a positive familial history for Type 2 Diabetes Mellitus) [16].

Although fasting glucose demonstrated a weaker association (cut-off 87.5 mg/dL, AUROC = 0.59), its inclusion remains clinically relevant as glucose intolerance often precedes overt hyperglycemia. Namely, we propose to all pediatric-centered clinicians to have a red flag of possible Insulin -resistance prior to end- organ disease onset at a fasting glucose level of 87.5 mg/dL. These specific children must be closely monitored for metabolic associated diseases onset. Collectively, these accessible and cost-effective parameters can be valuable additions to early IR screening, especially in resource-constrained settings.

We wish to emphasize that the AUROC values reported for lipid ratios and glucose, (~0.59–0.65) represent modest predictive accuracy and should not be overstated. Their main contribution lies in their accessibility and complementarity, rather than in providing stand-alone diagnostic precision.

A well-known predictive association is between visceral or central body fat accumulation and insulin-resistance derived metabolic disorders. As current anthropometric tools used to define excess body-fat disposition seem to fail in child and adolescent accurate prediction of metabolic derived comorbidities, emerging techniques for non-invasive body composition analysis are taken into account. With respect to better fat distribution mapping, body composition assessment was conducted in a part of our cohort using the Tanita PRO DC430 MA device. Resulting data was used to test different hypotheses regarding body-composition utility in IR prediction. So, BIA-derived variables—FFM, PMM, TBW, and WATERM—showed modest but comparable predictive power for IR prediction (AUROC~0.61) relative to biochemical markers. Although the predictive power of BIA-derived variables was modest, these findings still hold clinical relevance. While BIA alone may not provide definitive risk categorization, it contributes important body composition context, particularly in identifying sarcopenic obesity and TOFI phenotypes [17,18]. The ability to detect metabolically unhealthy normal-weight children further validates BIA’s role in supplementing BMI-based classification. Different fat depots exert distinct metabolic risks: visceral and ectopic fat have a stronger adverse impact on insulin sensitivity compared to subcutaneous fat, even in children with similar BMI [19]. These results support the integration of BIA in endocrine and pediatric obesity clinics, particularly for early phenotypic stratification. Our findings of modest discriminatory capacity of BIA-derived indices are consistent with previous pediatric studies reporting similar performance of fat-free mass and total body water in predicting insulin resistance [20]. Other groups have also shown that while BIA adds value in phenotypic stratification, its predictive power is not superior to traditional biochemical markers [21]. These convergent results reinforce the notion that BIA should be integrated as a complementary, rather than standalone, tool in pediatric obesity assessment.

An unexpected yet increasingly reported finding is the positive association between greater fat-free mass—including predicted muscle mass (PMM) and total body water (TBW)—and the presence of insulin resistance in pediatric populations. In this study, children with IR had significantly higher PMM, FFM, and TBW values compared to their insulin-sensitive counterparts, and these variables showed modest discriminatory capacity for IR (AUROC~0.61).

This seemingly paradoxical relationship has been observed in other studies and is thought to reflect the confounding effect of overall body size and adiposity—that is, children with higher fat mass tend to also have greater lean mass, but the metabolic burden is driven primarily by adiposity, not lean tissue quality per se [22,23]. Moreover, in the setting of sarcopenic obesity or low muscle quality, the protective effects of skeletal muscle on insulin sensitivity may be diminished despite normal or elevated muscle mass.

Furthermore, the type and distribution of lean mass matter: visceral fat infiltration of muscle, chronic low-grade inflammation, and reduced mitochondrial function may impair the insulin-sensitizing role of skeletal muscle [24,25]. Without direct measures of muscle function or strength (e.g., grip strength, physical activity level), bioimpedance-derived PMM may overestimate metabolically active muscle tissue.

Therefore, while PMM and FFM are higher in IR subjects, this should not be misinterpreted as protective. Instead, it reflects the increased total body mass in this group, underscoring the need to interpret lean mass indices alongside adiposity measures and metabolic markers. Future studies combining BIA with functional assessments (e.g., handgrip strength, VO_2_ max) or imaging (MRI, DXA) may clarify the role of muscle composition in pediatric insulin resistance.

As we advanced in the analysis of BIA-derived data, we aimed to determine whether the large volume of body composition variables could be simplified—retaining relevance while eliminating redundancy. We envisioned that reducing the number of dimensions would not only enhance daily clinical utility but also improve patients’ understanding of their body composition, which may support better adherence to management plans. To achieve this, we applied Principal Component Analysis (PCA) to the BIA data. The PCA identified two distinct and meaningful metabolic dimensions: a fat-free mass component (with TBW, WATERM, PMM, and FFM loading onto the first dimension) and an adiposity component (with WATERP, FATP, BMI, and FATM loading onto the second), together explaining over 90% of the variance.

These orthogonal axes reflect the heterogeneity of obesity phenotypes, from high-lean-mass individuals to those with disproportionate fat accumulation. Similar dimensional reductions have been reported in adolescent cohorts, supporting the biological relevance of separating lean and adipose compartments when assessing metabolic risk [21,26]. These components may also serve as early phenotypic markers in longitudinal studies, offering a novel framework for predicting metabolic trajectories and intervention outcomes. These mechanisms may also explain why some children already manifest intermediate cardiometabolic risk states such as prediabetes or early elevations in blood pressure despite their young age.

The novelty of our work rests primarily in the dimensional reduction in BIA data using PCA, which aligns with prior adolescent studies but, to our knowledge, has not yet been systematically applied in Eastern European pediatric populations. This approach highlights the heterogeneity of obesity-related phenotypes and offers a conceptual framework for future longitudinal risk stratification.

### Study Limitation

This study has several limitations that should be acknowledged. First, its retrospective design inherently limits control over data completeness and standardization. As a result, not all subjects underwent the same set of biochemical or anthropometric assessments, which restricted the inclusion of some parameters in the statistical analyses. Second, the heterogeneity in timing and indications for laboratory testing across the medical records may have introduced selection bias and reduced comparability between patients. Additionally, the sample size for certain sub-analyses—such as HbA1c levels or gonadotropic evaluations—was relatively small, further limiting the statistical power to detect subtle associations.

Beyond the retrospective design and incomplete data availability, additional limitations must be noted. First, the use of a universal HOMA-IR threshold (>2) does not fully reflect age- and puberty-specific physiology, and our exploratory ROC-derived cut-offs cannot be generalized without further validation in larger, prospective cohorts. Second, while BIA provides practical insights into body composition, it lacks the precision of gold-standard methods such as DXA or MRI. The associations observed between higher lean mass indices and insulin resistance likely reflect confounding by overall body size rather than muscle quality, which was not directly assessed. Third, the single-center, tertiary referral setting may limit generalizability to community populations.

Also, the TOFI subgroup comprised only five patients, with incomplete data for several variables. For this reason, no statistical comparisons with the rest of the cohort were performed. The findings are therefore presented descriptively as a signal-generating observation and should be interpreted with caution.

Nevertheless, we acknowledge that with a hospital-based sampling (potential Berkson selection) and retrospective design, our findings reflect an endocrino-pediatric referral population, not community prevalence. Taken together, these findings should be viewed as hypothesis-generating, forming a basis for future multicenter, longitudinal studies with standardized methodology.

## 5. Conclusions

This study reinforces the high proportion of insulin resistance (IR) in Romanian children with overweight and obesity, emphasizing the need for early identification, especially during puberty. Beyond traditional anthropometric markers, the study highlights the clinical utility of Tg/HDL ratio, fasting glucose, and bioelectrical impedance analysis (BIA)-derived parameters in detecting early metabolic risk. The novel PCA-based dimensional reduction in BIA data identified two distinct metabolic profiles—fat-free mass and adiposity—underlining the heterogeneity of pediatric obesity phenotypes. While higher lean mass in IR subjects may seem paradoxical, it likely reflects overall body mass rather than metabolic protection, reinforcing the importance of integrated interpretation of body composition and biochemical markers. As a pilot study, our findings should be interpreted with caution due to inherent limitations, including sample size and lack of longitudinal follow-up; nonetheless, they offer important preliminary insights that warrant confirmation in larger, multicenter cohorts. Together, these findings support a more nuanced, phenotype-driven approach to IR risk stratification in pediatric practice and advocate for the integration of accessible, non-invasive tools like BIA in early screening strategies.

## Figures and Tables

**Table 1:** Summary of Study Cohort Characteristics.

Biochemical and Anthropometric Variables	Values, Median (Minimum–Maximum)
Insulin uIU/mL (n = 206)	10.40 (0.20–138.10)
Fasting glucose, mg/dL (n = 208)	88 (66–116)
HOMA-IR (n = 204)	2.28 (0.03–34)
HbA1c % (n = 36)	5.72 (4.28–6.80)
ALT, U/L (n = 183)	27 (14–378)
Total Cholesterol, mg/dL (n = 184)	156 (60–265)
HDL-Cholesterol, mg/dL (n = 184)	45 (13–85)
LDL-Cholesterol, mg/dL (n = 184)	99.50 (12–193)
Triglycerides, mg/dL (n = 177)	83 (24–344)
Testosteron, ng/mL (n = 119)	0.52 (0.05–6.21)
Estradiol, pg/mL (n = 102)	15.05 (0.9–186.30)
LH, IU/mL(n = 109)	2.1 (0.10–13.31)
TSH, uUI/mL(n = 177)	3.2 (0.05–12.17)
Height, cm (n = 127)	157 (114–183)
Weight, kg (n = 127)	68 (27.1–145.5)
FATP, % (n = 127)	36.7 (18.3–62)
FATM, kg (n = 127)	15.36 (7.8–90.1)
FFM, kg(n = 127)	43.5 (19.3–75.6)
TBW, kg (n =127)	31.8 (14.1–55.3)
PMM, % (n = 127)	41.3 (18.2–71.9)
BMI, kg/mp (n = 127)	27.4 (19.6–55.3)
WATERM, kg (n = 127)	31.8 (14.1–55.3)
WATERP, %(n = 127)	46.4 (27.83–59.83)
BMI Percentile (n = 127)	98 (71–150)
Tg/HDL Ratio (n = 177)	2 (1–9.8)
FFM/FATM Ratio (n = 127)	1.72 (0.16–4.46)

Abbreviations: n = total number, HOMA-IR = Homeostatic Model Assessment of Insulin Resistance; HbA1c = glycated hemoglobin, ALT = alanine transaminase, HDL = high density lipoprotein, LDL = low density lipoprotein, LH = luteinizing hormone, TSH = Thyroid-stimulating hormone, FATP = fat mass percentage, FATM = total fat mass, FFM = free fat mass, TBW = total body water, PMM = predicted muscle mass, BMI = body mass index, WATERM = total water mass, WATERP = water mass percentage, Tg/HDL = triglycerides to HDL ratio.

**Table 2:** Summary of TOFI Subgrup Characteristics.

TOFI Subgrup	n = 5
Gender (female)	3/5
Median age (years)	15.5 (13–17)
Insulin resistance proportion	3/5
Median fasting blood glucose (mg/dL)	83 (78–98)
MASLD proportion	1/5
FATP, %	28.5 (18.3–32.4)
FATM, kg	16.8 (10.6–19.6)
FFM, kg	42.2 (38–59)
TBW, kg	30.9 (27.8–43.2)
PMM, %	40 (36–56)
BMI, kg/mp	21.7 (20.2–25.4)
WATERM, kg	30.9 (27.8–43.2)
WATERP, %	52.37(49.55–59.8)

Abbreviations: TOFI = Thin Outside, Fat Inside”, n = total number, FATP = fat mass percentage, FATM = total fat mass, FFM = free fat mass, TBW = total body water, PMM = predicted muscle mass, BMI = body mass index, WATERM = total water mass, WATERP = water mass percentage.

**Table 3:** Summary of ROC analyses for predictors of insulin resistance (HOMA-IR > 2) and obesity phenotypes.

Predictor	Outcome	AUROC (95% CI)	Optimal Cut-Off	Sensitivity (%)	Specificity (%)
Tg/HDL ratio	IR (HOMA-IR > 2)	0.645 (0.562–0.727)	1.789	60.0	58.2
Triglycerides (mg/dL)	IR (HOMA-IR > 2)	0.656 (0.576–0.737)	80	61.9	60.0
Fasting glucose (mg/dL)	IR (HOMA-IR > 2)	0.590 (0.519–0.674)	87.5	58.3	57.0
FFM (kg)	IR (HOMA-IR > 2)	0.607 (0.506–0.708)	42	63.4	57.1
TBW (kg)	IR (HOMA-IR > 2)	0.614 (0.514–0.715)	30.75	63.4	57.1
PMM (kg)	IR (HOMA-IR > 2)	0.617 (0.517–0.717)	39.85	63.4	57.1
WATERM (kg)	IR (HOMA-IR > 2)	0.612 (0.511–0.712)	30.75	63.4	57.1

Abbreviations: Tg/HDL ratio = triglycerides to HDL ratio, HDL = high density lipoprotein, FFM = free fat mass, TBW = total body water, PMM = predicted muscle mass, WATERM = total water mass.

**Table 4:** Correlation matrix of BIA parameters.

Variables	FATP	FATM	FFM	TBW	PMM	WATERM	WATERP	BMI
FATP	1.000	0.571	0.078	−0.001	0.004	0.006	−0.930	0.650
FATM	0.571	1.000	0.353	0.365	0.365	0.368	−0.606	0.716
FFM	0.078	0.353	1.000	0.970	0.952	0.957	0.018	0.627
TBW	−0.001	0.365	0.970	1.000	0.982	0.986	0.008	0.651
PMM	0.004	0.365	0.952	0.982	1.000	0.968	0.002	0.646
WATERM	0.006	0.368	0.957	0.986	0.968	1.000	−0.001	0.650
WATERP	−0.930	−0.606	0.018	0.008	0.002	−0.001	1.000	−0.692
BMI	0.650	0.716	0.627	0.651	0.646	0.650	−0.692	1.000

Abbreviations: BIA = Bioimpedance Analysis, FATP = fat mass percentage, FATM = total fat mass, FFM = free fat mass, TBW = total body water, PMM = predicted muscle mass, BMI = body mass index, WATERM = total water mass, WATERP = water mass percentage.

## Data Availability

The data presented in this study are available in the Supplementary Materials of this article.

## Supplementary Materials

The following supporting information can be downloaded at: https://www.mdpi.com/article/10.3390/medicina61091709/s1, Dataset S1: Anonymized dataset containing the variables used for analysis in this study.
